# Occlus-o-Guide^® ^*versus *Andresen activator appliance: neuromuscular evaluation

**DOI:** 10.1186/2196-1042-14-4

**Published:** 2013-05-20

**Authors:** Giampietro Farronato, Lucia Giannini, Guido Galbiati, Elena Grillo, Cinzia Maspero

**Affiliations:** 1 Department of Orthodontics, Fondazione IRCCS Cà Granda - Ospedale Maggiore Policlinico, University of Milan, Milan 20122, Italy

**Keywords:** Functional appliances, Occlus-o-Guide^®^, Activator, Electromyography

## Abstract

**Background:**

The aim of the present study was to assess the muscular variations at the electromyography (EMG) level for the anterior temporalis muscles and masseter muscles during treatment with Occlus-o-Guide^®^ and Andresen activator appliances.

**Methods:**

Eighty-two patients (35 males and 47 females) aged between 8 and 12 years (mean age, 10.5 ± 0.8 years) participated in the study. Fifty patients underwent treatment with an Occlus-o-Guide^®^ and 32 patients with an Andresen activator. All patients underwent EMG examination using a Freely EMG (De Gotzen, Legnano, Italy) and surface bipolar electrodes when the appliances were worn for the first time (T0), and after 6 months (T1) and after 12 months (T2) of appliance use.

**Results:**

Statistical analysis showed that both at T0 and T2, the percent overlapping coefficient (POC) of the anterior temporalis muscles was not statistically different between the appliance groups. At T0, the POC of the masseter muscles was significantly lower for the Andresen appliance as compared to the Occlus-o-Guide^®^ (*p* = 0.02), while at T2 this significance was lost.

**Conclusions:**

At insertion of an appliance, all patients show neuromuscular balance that does not correspond to orthognathic occlusion. Both appliances work by creating muscular imbalance. With the appliances *in situ*, EMG responses were generally analogous for the Occlus-o-Guide^®^ and the Andresen activator; however, the imbalance was greater and the recovery of the orthological muscular balance was slower in patients under treatment with the Andresen activator as compared to those with the Occlus-o-Guide^®^.

## Background

In recent years, technological innovations have led to the production and introduction into clinical orthodontic practice of electronic instruments for the recording of patient physiological and biological data obtainable through objective examinations. These now also supply diagnostic documents that have medico-legal value [1-4]. Surface electromyography (EMG) represents one of these instrumental techniques [1].

Functional orthodontic appliances used with growing patients have important roles relating to neuromuscular function. The use of EMG allows the analysis of the neuromuscular patterns of patients before, during and after therapy with such functional appliances.

Many authors have highlighted the effects of functional orthodontic appliances on the stomatognathic systems [5-10]. Ahlgren [6] indicated that the protractor muscles of the mandible are stimulated during daytime use of activators while the retractor muscles are inhibited; these effects are not seen during night-time use. Ahlgren [6] also showed that before and after activator treatment, Class II patients have balanced EMG patterns.

Sander [11] also described different effects of functional therapies between day and night use, whereby wearing an appliance during the day corresponds to neuromuscular programming. Aggarwal et al. [12] showed that in Class II patients there is a significant increase in EMG activity in the masseter and anterior temporalis muscles during treatment with a twin block, due to an enhanced stretch reflex of the elevator muscles. Indeed, in patients in therapy with an activator, the viscoelasticity of the soft tissues generates a passive tension that has a more important role than the phasic stretch reflex during orthopaedic therapy with activators [13]. Yuen et al. [14] also showed that the effects of an activator on the neuromuscular system are due to the downward shifts of the mandible, which change the fibre lengths of the muscles.

At the beginning of treatment with an activator, Uner et al. [15] showed evidence for an increase in the activities of the masseter and temporalis muscles in the rest position and a decrease in the maximum biting force. At the end of the treatment, the activities of both muscles had decreased in the rest position. Both changes were recorded during EMG only with the activator positioned in the mouth of the patients; no changes were seen without the activator in position.

**Figure 1:**
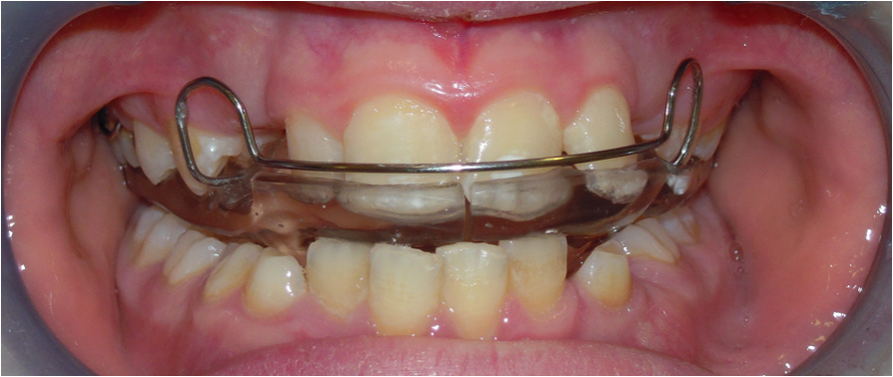
Intraoral frontal view of a patient wearing an Occlus-o-Guide^® ^ appliance

**Figure 2:**
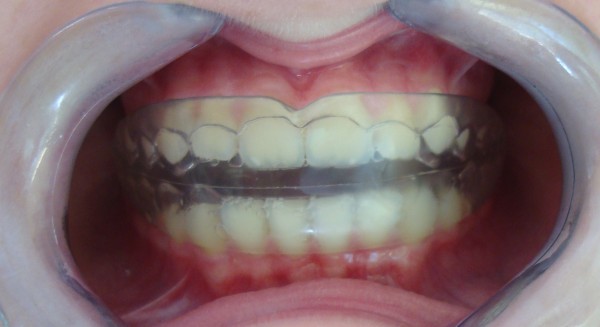
Intraoral frontal view of a patient wearing an Andresen activator.

The present study illustrates the clinical applications of EMG in orthodontics and emphasises how the use of this diagnostic method allows basic information to be obtained with respect to the functional needs for malocclusion correction. Furthermore, results obtained from an EMG study in patients under treatment with functional appliances are also shown, relating to the use of an Occlus-o-Guide^®^ (Ortho-Tain 950 Green Bay Road Winnetka, IL 60093) and an Andresen activator (Figures 1 and 2, respectively). The aim of the present study was, therefore, to assess the muscular variations at the EMG level for the anterior temporalis muscles and masseter muscles during treatment with Occlus-o-Guide^®^ and Andresen activator appliances.

## Methods

Eighty-two patients (35 males and 47 females) aged between 8 and 12 years (mean age, 10.5 ± 0.8 years) participated in the present study. Fifty patients underwent treatment with an Occlus-o-Guide^®^ and 32 patients with an Andresen activator.

The selection criteria for patient participation were as follows:

(a) Skeletal and dental Class II malocclusion (divisions 1 and 2).

(b) Skeletal and dental deep bite.

(c) Carpal growth index in period IV [4], pubertal spurt.

(d) No temporomandibular joint disorders.

(e) Absence of periodontal disease.

Initial impressions were taken from all of the patients, and cephalometric tracings were performed. All of the cephalometric tracings were carried out by the same operator (C.M.) to minimise measurement errors. The selected sample turned out to be homogeneous for skeletal age (timing) and malocclusion. All patients during the pubertal growth spurt were asked to give maximum cooperation.

All of the patients for treatment with the Occlus-o-Guide^®^ received either a G-type or N-type appliance, according to their dentition phase. G-type appliances are indicated for malocclusions in the mixed dentition, and they guide posterior teeth eruption into a Class I relationship. N-type appliances are indicated in permanent dentition, where the design prevents overbite relapse while at the same time advancing the mandible. The patients were instructed to use the devices by gradually increasing the application period up to 2 to 4 h during the day, plus all night.

An Andresen-type appliance was constructed for each candidate for treatment with an activator, using construction wax taken in an incisor edge-to-edge position or otherwise within the maximum protrusion of the patient. Resin capping was applied to the activator on the labial side of both the upper and lower front teeth to reduce the vestibular inclination effect that has been documented in the literature [16].

All of the patients underwent EMG examination using a Freely EMG (De Gotzen, Legnano, Italy) and surface bipolar electrodes. All of the EMG examinations were carried out by the same operator (L.G.) to minimise measurement errors. For every patient, three datapoints were included: when the appliances were worn for the first time (T0), and after 6 months (T1) and 12 months (T2) of appliance use. The patients were asked to repeat every exercise three times for every datapoint, with the mean used. A total of 1,530 tests were carried out.

During each data acquisition, the patient carried out the tests provided for the EMG protocol as detailed by the Laboratory of Functional Anatomy of the Stomatognathic System (*Laboratorio di Anatomia Funzionale dell’Apparato Stomatognatico*): cotton, clench, clench-rest and tap tests. Subsequently, the patients were asked to wear the appliance and to exert maximum voluntary contraction on the appliance for 5 s, without making the head shake or the face wrinkle so as not to affect the test results.

The EMG data analysed included the percent overlapping coefficient (POC) of the anterior temporalis muscle (POC-ATM) and the masseter muscle (POC-MM), and the clench measures as percentages of muscular contraction of the right/left anterior temporalis muscles (%RATM/%LATM) and of the right/left masseter muscles (%RMM/%LMM). These were all calculated from the values obtained with clenching on the teeth divided by those obtained with clenching on cotton rolls, expressed as percentages. The distributions of the clinical variables between the two groups at each data collection point were compared using the Mann–Whitney test. Statistical analyses were performed with Stata 11 (StataCorp LP, College Station, TX, USA). All the patients signed an informed consent to partecipate at the study. No ethical approval was necessary because actually the electromyographic tests were requested to complete the diagnostic process.

## Results

Without the appliances in place, the indices for the POC-ATM and the POC-MM always showed values ≥85% in the clench tests for all of the datapoint acquisitions in the EMG tests (Table 1). In contrast, with the appliances *in situ*, these values were increased from 79% to 86%, with the POCs for both appliances tending to increase during the treatment, from T0 to T2.

**Table 1:** EMG clench measures while not wearing the appliances

**EMG measure without appliances**	**Appliance used**	**EMG examination datapoint**		
		**T0**	**T1**	**T2**
POC-ATM (%)	Occlus-o-Guide^®^	87 ± 5	87 ± 3	87 ± 4
	Andresen activator	89 ± 2	87 ± 7	88 ± 5
	*p*	0.16	0.82	0.05
POC-MM (%)	Occlus-o-Guide^®^	86 ± 5	86 ± 6	88 ± 4
	Andresen activator	88 ± 3	85 ± 6	88 ± 3
	*p*	0.19	0.68	0.88
%RATM	Occlus-o-Guide^®^	112 ± 27	105 ± 22	101 ± 24
	Andresen activator	112 ± 30	108 ± 21	108 ± 28
	*p*	0.85	0.09	0.13
%LATM	Occlus-o-Guide^®^	113 ± 24	109 ± 24	107 ± 22
	Andresen activator	112 ± 25	116 ± 23	108 ± 28
	*p*	0.71	0.18	0.90
%RMM	Occlus-o-Guide^®^	102 ± 27	101 ± 25	102 ± 18
	Andresen activator	111 ± 34	112 ± 24	109 ± 25
	*p*	0.53	0.01	0.15
%LMM	Occlus-o-Guide^®^	105 ± 27	104 ± 30	104 ± 21
	Andresen activator	111 ± 24	115 ± 36	109 ± 30
	*p*	0.30	0.15	0.97

Data are means ± standard deviation. T0, first use; T1, after 6 months; T2, after 12 months; POC-ATM, percent overlapping coefficient of the anterior temporalis muscles; POC-MM, percent overlapping coefficient of the masseter muscles; %RATM, %LATM, %RMM, %LMM, clench percentages for each muscle; *p*, significance between appliances (Mann–Whitney test).

When the patients were not wearing the appliances, the muscular contractions in the clench tests were constantly >100% (Table 1), while when they were wearing the appliances, these values were lower (Table 2). These values also showed their maximum inhibition on delivery (T0) and gradually recovered during the treatments.

**Table 2:** EMG clench measures while wearing the appliances

**EMG measure with appliance**	**Appliance used**	**EMG examination datapoint**		
		**T0**	**T1**	**T2**
POC-ATM (%)	Occlus-o-Guide^®^	79 ± 13	82 ± 7	83 ± 4
	Andresen activator	73 ± 17	81 ± 10	82 ± 16
	*p*	0.25	0.72	0.09
POC-MM (%)	Occlus-o-Guide^®^	84 ± 8	85 ± 7	86 ± 4
	Andresen activator	80 ± 8	84 ± 6	84 ± 12
	*p*	0.02	0.05	0.11
%RATM	Occlus-o-Guide^®^	60 ± 29	64 ± 19	68 ± 23
	Andresen activator	46 ± 27	68 ± 36	88 ± 37
	*p*	0.05	0.96	0.001
%LATM	Occlus-o-Guide^®^	66 ± 39	65 ± 18	71 ± 26
	Andresen activator	52 ± 28	58 ± 26	86 ± 24
	*p*	0.10	0.04	0.009
%RMM	Occlus-o-Guide^®^	87 ± 36	89 ± 26	99 ± 36
	Andresen activator	60 ± 30	90 ± 32	99 ± 29
	*p*	0.001	0.92	0.40
%LMM	Occlus-o-Guide^®^	85 ± 43	88 ± 24	95 ± 31
	Andresen activator	60 ± 30	87 ± 29	98 ± 32
	*p*	0.005	0.77	0.16

Data are means ± standard deviation. T0, first use; T1, after 6 months; T2, 12 after months; POC-ATM, percent overlapping coefficient of the anterior temporalis muscles; POC-MM, percent overlapping coefficient of the masseter muscles; %RATM, %LATM, %RMM, %LMM, clench percentages for each muscle; *p*, significance between appliances (Mann–Whitney test).

The neuromuscular imbalance caused by the use of the Andresen appliance was greater when compared to that of the Occlus-o-Guide^®^ because with the appliances in place, in all of the data acquisitions, the POC-ATM and POC-MM were always lower for the Andresen appliance (although not necessarily statistically significant). Statistical analysis showed that both at T0 and T2, the POC-ATM index was not statistically different between the appliance groups (Table 2, *p* = 0.25, *p* = 0.09, respectively). At T0, the POC-MM was significantly lower for the Andresen appliance as compared to the Occlus-o-Guide^®^ (Table 2, *p* = 0.02), while at T2 this significance was lost (Table 2, *p* = 0.11). The left temporal index (%LATM) was generally lower for the Andresen appliance, with this difference from the Occlus-o-Guide^®^ reaching significance both at T1 and T2 (Table 2, *p* = 0.04, *p* = 0.009, respectively); the right temporal index (%RATM) was only significantly smaller for the Andresen appliance at T2 as compared to the Occlus-o-Guide^®^ (Table 2, *p* = 0.001). No statistically significant differences were found for the Andresen appliance with the right masseter index (%RMM) and left masseter index (%LMM) as compared to the Occlus-o-Guide^®^, except for the values at T0 (Table 2, *p* = 0.001, *p* = 0.005, respectively).

## Discussion

Mandibular growth can be influenced by a variety of functional orthodontic appliances because of the skeletal and neuromuscular adaptations that occur as a response to therapy [14-24].

For this reason, a lot of studies have investigated the muscle changes during such functional treatment [17-32]. Hiyama et al. analysed neuromuscular adaptation to functional therapy through the use of needle EMGs [17]. Indeed, functional orthodontic appliances have been used for decades to correct skeletal malocclusions. Clinicians have suggested that the changes associated with functional appliances are due to the enhancement of muscular activity when dramatic skeletal and occlusal changes occur [29]. This, in turn, can modify mandibular and maxillary growth while guiding the eruption of teeth into more acceptable relationships [33]. However, it is still unclear how functional appliances influence the jaw muscles and mediate bony changes [34-36].

In the present study, without the appliances in place, for the patients under treatment with both the Occlus-o-Guide^®^ and the Andresen appliance, the percentages of muscular contraction during the clench test for both the anterior temporalis muscles and the masseter muscles were >100% before and after the treatments. On the basis of what has been said, it can thus be deduced that these patients under treatment with both the Occlus-o-Guide^®^ and the Andresen appliance should show neuromuscular balance benefit when carrying out the test of maximum voluntary clench on their teeth, over the period of the whole treatment. However, their condition worsened when the test was carried out with the appliances placed inside their mouths, with maximum inhibition at T0, but with a constant recovery through T1 to T2.

Compared with those with the Occlus-o-Guide^®^, the patients with the Andresen activator showed contraction values with the activators in place that were lower at T0. This arises as the Andresen activator has a customised construction bite while the Occlus-o-Guide^®^ has a standard one. Thus, the neuromuscular imbalance caused by the activator is apparently greater due to this customised construction bite.

These results are in agreement with the study of Ahlgren [6], who demonstrated that in Class II patients the protractor muscles are weak and hypotonic. In this study, Ahlgren [6] analysed 20 Class II cases treated with activators, noting that (1) during daytime use of activators, the retractor muscles of the mandible are inhibited while the protractor muscles are stimulated, and no functional stimulation could be shown; (2) before and after the treatment, the Class II cases show a balanced EMG pattern during closure in the intercuspal position; and (3) a narrow maxillary arch should be expanded before treatment to make it easier for the lower arch to adapt itself to a protruded position. Ahlgren [6] also found the same results in his studies of EMG responses during therapy with an activator. Of note, the much earlier study by Eschler [34] found different results in Class II patients, where he reported that the activator stimulated the retractor muscles by the stretching reflex.

Regular evaluation of patients under orthodontic treatment from a neuromuscular point of view adds functional evaluation to clinical practice. Furthermore, it makes it possible to instrumentally obtain an evaluation of some parameters that would otherwise be difficult to evaluate, if not empirically. The role of the musculature in the diagnostic-therapeutic field is becoming more and more important, particularly as this is often the subject of medico-legal disputes.

It can be confirmed that the masticatory muscles respond positively to treatments with elastodontic appliances when they are used to bring the patient from compensatory balance to orthological balance. Indeed, during functional therapy, the elevator muscles undergo an elongation that is proportional to the amount of bite raising and mandibular protrusion [34]. When a muscle changes its length, the shape and amplitude of the motor unit change progressively [14,35]. Before structural adaptation to the masseter muscle, this lengthening takes place, and an increased activity would act to restore the original length reflexively. This adaptation of the muscular function takes place within a relatively short period, before compensatory morphological changes can occur [7].

An increase in the postural activity of the superior head of the lateral pterygoid muscle after the insertion of a functional appliance might be responsible for the increased condylar growth in young animals, as suggested by the lateral pterygoid muscle hypothesis. Increased jaw elevator muscle activity during swallowing is necessary to stabilise the lower jaw against the appliance [26,27,36]. On the other hand, previous EMG investigations carried out in non-human primates have indicated that the application of jaw-protruding functional appliances promotes a decrease in the function of these muscles, instead of an increase [37]. Sessle et al. [37] monitored the activity of the masticatory muscles with chronically implanted EMG electrodes to determine whether such functional appliances produce a change in postural EMG activity of the muscles. They concluded that the insertion of two types of functional appliance to induce mandibular protrusion was associated with a decrease in the postural EMG activity of the superior and inferior heads of the lateral pterygoid, superficial masseter and anterior digastric muscles [37].

After insertion of an appliance into the mouth, this might also promote a change in swallowing patterns. After appliance insertion, different facultative muscles, such as the facial muscles, can contribute to swallowing, with a consequent decrease in the activity in the other muscles [29,38-41].

## Conclusions

1. Upon insertion of an appliance (T0), all of the patients showed neuromuscular balance that did not correspond to orthognathic occlusion, both for those under treatment with the Occlus-o-Guide^®^ and those under treatment with the Andresen activator.

2. Both appliances work by creating muscular imbalance, as documented by the variations in the EMG indices and the maximum imbalance recorded in the first 6 months of treatment.

3. With the appliances *in situ*, the EMG responses were basically analogous for the Occlus-o-Guide^®^ and the Andresen activator. However, the imbalance was greater and the recovery of the orthological muscular balance was slower in patients under treatment with the Andresen activator as compared to those with the Occlus-o-Guide^®^.

The masticatory muscles (anterior temporalis and masseter muscles) respond positively to treatment with elastodontic appliances in the attempt to bring a patient from compensatory balance to orthological balance. We believe that a step forward in the future will be a compromise between these two appliances, which will need to combine the clinical applications of the Andresen activator with the resilience characteristics and the clinical applications of the eruption guide of the Occlus-o-Guide^®^.

## Competing interests

The authors declare that they have no competing interests.

## Authors’ contributions

All authors actively participated to all phases of the manuscript and in treating patients. All authors read and approved the final manuscript.

## References

[B1] Tartaglia GM da Moreira Rodrigues SMA Bottini S Sforza C Ferrario VF Masticatory muscle activity during maximum voluntary clench in different research diagnostic criteria for temporomandibular disorders (RDC/TMD) groups Man Ther 2008 13 434 40 10.1016/j.math.2007.05.011 17643338

[B2] Mortellaro C Rimondini L Farronato G Garagiola U Varcellino V Berrone M Temporomandibular disorders due to improper surgical treatment of mandibular fracture: clinical report J Craniofac Surg 2006 17 373 82 10.1097/00001665-200603000-00032 16633194

[B3] Sforza C Peretta R Grandi G Ferronato G Ferrario VF Soft tissue facial planes and masticatory muscle function in skeletal class III patients before and after orthognathic surgery treatment J Oral Maxillofac Surg 2008 66 691 8 10.1016/j.joms.2007.06.645 18355592

[B4] Farronato G Giannini L Galbiati G Sesso G Maspero C Orthodontic-surgical treatment: neuromuscular evaluation in skeletal Class II and Class III patients Prog Orthod 2012 13 3 226 36 10.1016/j.pio.2012.04.003 23260533

[B5] Pancherz H Anehus M Masticatory function after activator treatment. An analysis of masticatory efficiency, occlusal contact conditions and EMG activity Acta Odontol Scand 1978 36 309 16 10.3109/00016357809029081 281105

[B6] Ahlgren J Early and late electromyographic response to treatment with activators Am J Orthod 1978 74 88 93 10.1016/0002-9416(78)90048-9 278485

[B7] Carels C van Steenberghe D Changes in neuromuscular reflexes in the masseter muscles during functional jaw orthopedic treatment in children Am J Orthod Dentofacial Orthop 1986 90 410 9 10.1016/0889-5406(86)90006-5 2946221

[B8] Palmieri A Zollino I Clauser L Lucchese A Girardi A Farinella F Carinci F Biological effect of resorbable plates on normal osteoblasts and osteoblasts derived from Pfeiffer syndrome J Craniofac Surg 2011 22 3 860 3 10.1097/SCS.0b013e31820f7d34 21558934

[B9] Lucchese A Carinci F Brunelli F Skeletal effects induced by twin block in therapy of class II malocclusion Eur J Inflamm 2012 10 S1 83 6

[B10] Farronato G Giannini L Galbiati G Maspero C Sagittal and vertical effects of rapid maxillary expansion in Class I, II, and III occlusions Angle Orthod 2011 81 2 298 303 10.2319/050410-241.1 21208083 PMC8925266

[B11] Sander FG Functional processes when wearing the SII appliance during the day J Orofac Orthop 2001 62 264 74 10.1007/PL00001934 11508103

[B12] Aggarwal P Kharbanda OP Mathur R Duggal R Parkash H Muscle response to the twin-block appliance: an electromyographic study of the masseter and anterior temporal muscles Am J Orthod Dentofacial Orthop 1999 116 405 14 10.1016/S0889-5406(99)70225-8 10511668

[B13] Noro T Tanne K Sakuda M Orthodontic forces exerted by activators with varying construction bite heights Am J Orthod Dentofacial Orthop 1994 105 169 79 10.1016/S0889-5406(94)70113-X 8311039

[B14] Yuen SW Hwang JC Poon PW Changes in power spectrum of electromyograms of masseter and anterior temporal muscles during functional appliance therapy in children Am J Orthod Dentofacial Orthop 1990 97 301 7 10.1016/0889-5406(90)70102-I 2321596

[B15] Uner O Darendeliler N Bilir E Effects of an activator on the masseter and anterior temporal muscle activities in Class II malocclusions J Clin Pediatr Dent 1999 23 327 32 10551133

[B16] Farronato G Giannini L Galbiati G Maspero C Long term results of open reduction management of condylar fracture: a 20 years follow-up Case report. Minerva Stomatol 2012 Oct 61 10 457 65 23076028

[B17] Hiyama S Ono PT Ishiwata Y Kuroda T McNamara JA Jr Neuromuscular and skeletal adaptations following mandibular forward positioning induced by the Herbst appliance Angle Orthod 2000 70 442 53 11138648 10.1043/0003-3219(2000)070<0442:NASAFM>2.0.CO;2

[B18] Graber TM Neumann B Graber TM, Neumann B Functional orthopedics - its concept and transition Removable Orthodontic Appliances 1984 Tokyo: Ishiyaku 103 17

[B19] Neumann B Graber TM Removable appliances Current Orthodontic Concepts and Techniques 1969 Philadelphia: WB Saunders 817 74

[B20] Field HW Proffit WR Treatment of skeletal problems in preadolescent children Contemporary Orthodontics 1986 St. Louis: CV Mosby 357

[B21] Andresen V The Norwegian system of functional gnatho-orthopedics Acta Gnathol 1936 1 5 36

[B22] Frankel R The treatment of Class II, division 1 malocclusion with functional correctors Am J Orthod 1969 55 265 75 10.1016/0002-9416(69)90106-7 5250511

[B23] Pancherz H Treatment of class II malocclusions by jumping the bite with the Herbst appliance Am J Orthod 1979 76 423 42 10.1016/0002-9416(79)90227-6 291343

[B24] Pancherz H The mechanism of class II correction in Herbst appliance treatment - a cephalometric investigation Am J Orthod 1982 82 104 13 10.1016/0002-9416(82)90489-4 6961781

[B25] Pancherz H The Herbst appliance - its biologic effects and clinical use Am J Orthod 1985 87 1 20 10.1016/0002-9416(85)90169-1 3855346

[B26] McNamara JA Jr Howe RP Dischinger TG A comparison of the Herbst and Frankel appliances in the treatment of class II malocclusion Am J Orthod Dentofacial Orthop 1990 98 134 44 10.1016/0889-5406(90)70007-Y 2378319

[B27] Mills CM McCulloch KA Treatment effect of the twin block appliance: a cephalometric study Am J Orthod Dentofacial Orthop 1998 114 15 24 10.1016/S0889-5406(98)70232-X 9674675

[B28] McNamara JA Jr Neuromuscular and skeletal adaptations to altered function in the orofacial region Am J Orthod 1973 64 578 606 10.1016/0002-9416(73)90290-X 4210182

[B29] Yamin-Lacouture C Woodside DG Sectakof PA Sessle BJ The action of three types of functional appliances on the activity of the masticatory muscles Am J Orthod Dentofacial Orthop 1997 112 560 72 10.1016/S0889-5406(97)70102-1 9387844

[B30] Woodside DG Metaxas A Altuna G The influence of functional appliance therapy on glenoid fossa remodeling Am J Orthod Dentofacial Orthop 1987 92 181 98 10.1016/0889-5406(87)90411-2 3477085

[B31] Miralles R Berger B Bull R Manns A Carvajal R Influence of the activator on electromyographic activity of mandibular elevator muscles Am J Orthod Dentofacial Orthop 1988 94 97 103 10.1016/0889-5406(88)90357-5 3165246

[B32] Nucci P Farronato G Serafino M Brusati R Restrictive strabismus after blow-out orbital fracture in children: is the muscle involved? J Trauma 2004 56 1 209 10 10.1097/01.TA.0000096512.45631.AF 14749594

[B33] der Maur HJA Electromyographic recordings of the lateral pterygoid muscle in activator treatment of Class II, division 1 malocclusion cases Eur J Orthod 1980 2 161 71 10.1093/ejo/2.3.161 6935066

[B34] Eschler J Die funktionelle Orthopaedie des Kausystems 1952 Munchen, Hanser

[B35] Du X Hagg U Muscular adaptation to gradual advancement of the mandible Angle Orthod 2003 73 525 31 14580019 10.1043/0003-3219(2003)073<0525:MATGAO>2.0.CO;2

[B36] Petrovic AG Postnatal growth of bone: a perspective of current trends, new approaches, and innovations Prog Clin Biol Res 1982 101 297 331 6961464

[B37] Sessle BJ Woodside DG Bourque P Gurza S Powell G Voudouris J Metaxas A Altuna G Effect of functional appliances on jaw muscle activity Am J Orthod Dentofacial Orthop 1990 98 222 30 10.1016/S0889-5406(05)81599-9 2403073

[B38] Miller AJ Vargervik K Chierici G Electromyographic analysis of the functional components of the lateral pterygoid muscle in the rhesus monkey (*Macaca mulatta*) Arch Oral Biol 1982 27 475 80 10.1016/0003-9969(82)90087-5 6956260

[B39] Farronato G Carletti V Maspero C Farronato D Giannini L Bellintani C Craniofacial growth in children affcted by juvenile idiopathic arthritis involving temp. Joints: functional therapy management. Journal Clin Pediatric Dent 2009 33 4 351 7 10.17796/jcpd.33.4.05287m400q508772 19725245

[B40] Bellintani C Ghiringelli P Gerloni V Gattinara M Farronato G Fantini F Temporomand. Joint involvement in juvenile idiopathic arthritis: treatment with an orth. Appliance. Reumatismo 2005 57 3 201 7 16258606 10.4081/reumatismo.2005.201

[B41] Farronato G Garagiola U Carletti V Cressoni P Bellintani C Psoriatic Arthritis: temporomandibular joint involvement as the first articular phenomenon Quintessence International 2010 41 5 395 8 20376375

